# Fundamental privacy rights in a pandemic state

**DOI:** 10.1371/journal.pone.0252169

**Published:** 2021-06-02

**Authors:** Tânia Carvalho, Pedro Faria, Luís Antunes, Nuno Moniz

**Affiliations:** 1 Department of Computer Science, Faculty of Sciences, University of Porto, Porto, Portugal; 2 TekPrivacy, Porto, Portugal; 3 INESC TEC, Porto, Portugal; University of Management and Technology, PAKISTAN

## Abstract

Faced with the emergence of the Covid-19 pandemic, and to better understand and contain the disease’s spread, health organisations increased the collaboration with other organisations sharing health data with data scientists and researchers. Data analysis assists such organisations in providing information that could help in decision-making processes. For this purpose, both national and regional health authorities provided health data for further processing and analysis. Shared data must comply with existing data protection and privacy regulations. Therefore, a robust de-identification procedure must be used, and a re-identification risk analysis should also be performed. De-identified data embodies state-of-the-art approaches in Data Protection by Design and Default because it requires the protection of direct and indirect identifiers (not just direct). This article highlights the importance of assessing re-identification risk before data disclosure by analysing a data set of individuals infected by Covid-19 that was made available for research purposes. We stress that it is highly important to make this data available for research purposes and that this process should be based on the state of the art methods in Data Protection by Design and by Default. Our main goal is to consider different re-identification risk analysis scenarios since the information on the intruder side is unknown. Our conclusions show that there is a risk of identity disclosure for all of the studied scenarios. For one, in particular, we proceed to an example of a re-identification attack. The outcome of such an attack reveals that it is possible to identify individuals with no much effort.

## Introduction

On the last day of December 2019, the first reports of the Coronavirus disease, also known as Covid-19 [1], emerged in China. This virus is highly contagious and is mostly transmitted in humans by aerosols [2]. The Covid-19 outbreak quickly spread across the world, leading to an increase of people infected by the virus, and consequently deaths. For this reason, the World Health Organization declared the Covid-19 a pandemic [3]. To cooperate in combating the Coronavirus, several scientists from different fields contribute daily with their skills and expertise. One of the contributions to contain the disease is data analysis, which helps gain a more comprehensive understanding of it.

In this context, health organisations need to share data with organisations responsible for analysing the data. However, before sharing, they must ensure that all private information concerning each individual is de-identified. A de-identification procedure consists of removing or obfuscating personal information from a record or a data set. The re-identification, also known as identity disclosure, of individuals can occur by comparing de-identified data with other external data sources, such as public information available on social networks, websites, or other data sets. For example, researchers have recently revealed that supposedly de-identified data can easily be re-identified so that sensitive information is linked back to an individual [4, 5].

In this paper, we discuss re-identification risk and loss of privacy. Then, we analyse such factors given a data set generated by the Directorate-General of Health in Portugal (Direção Geral de Saúde) concerning Covid-19 cases, from January to mid-April 2020. The data was subject to a request for an opinion from the Ethics Committee and was approved on 16 April 2020 (Ref. Proc. CE2020/0401). Subsequently, and for demonstrative purposes only, we link the provided data set to an external data set. We analyse and discuss results, providing recommendations for best practices in releasing data sets in similar contexts to those of the studied case. At the end of this article, we provide conclusions and final remarks.

### Privacy in the scope of human rights and dignity

At a time of a pandemic affecting the entire world, the United Nations recalls the importance of the dignity and human rights of all citizens [6]. Also, the European Union (EU) recognises the principle of human dignity as an absolute fundamental right [7]. The right to privacy is one of these fundamental rights, and its basic premise is to serve as mechanisms capable of protecting individual dignity. In addition, both the Universal Declaration of Human Rights (Art. 12.°) [8], the European Convention on Human Rights (Art. 8.°) [9] and the Charter of Fundamental Rights of the European Union (Art. 7.°) [10], enshrine our right to privacy.

Privacy is a concept associated with confidentiality and includes the protection of information, particularly the personal data of individuals. Therefore, individuals’ protection concerning personal data processing is a fundamental right (Art. 8.°) [10], and their privacy is an essential factor.

Data protection aims to ensure legitimate processing of personal data by the public and private sectors [7]. Consequently, the processing of personal data must be lawful and proportionate to the purpose for which it was collected.

The General Data Protection Regulation (GDPR) (Regulation (EU) 2016/679 of the European Parliament and of the Council of 27 April 2016) emerged to unify data privacy laws across Europe. Its objective is to protect the private data of EU citizens and to reshape the way organisations approach data protection. The GDPR (Art. 25.°) [11] introduces two new concepts, Privacy by Design and Privacy by Default which is a requirement for data controllers. Privacy by Design requires data controllers to establish measures to implement data protection principles and incorporate the necessary safeguards to meet the regulation requirements and protect the rights of data subjects. On the other hand, Privacy by Default requires the controller to implement appropriate measures to ensure that only personal data needed are processed by default for the specific purpose of processing.

Any breach of personal data can compromise the privacy of individuals. For this reason, data controllers must inform individuals if a breach is likely to cause harm [12].

### Re-identification risk

The sharing of health data for secondary uses has increased, mainly for research [13]. An “obstacle” to sharing such data has been a concern for the privacy of individuals. In Portugal, the Portuguese Parliament ensures the execution, in the national legal order, of the GDPR. According to the law applied in Portugal, the de-identification of data is a process to protect the privacy of individuals [14]. Although the study is carried out at the Portugal level, we stress that this is a global problem, it is not exclusive only to Portugal or the European Union.

The procedure to protect individuals’ privacy begins with the removal of explicit identifiers such as name and social security numbers. However, when we combine other attributes, these can generate a unique combination, and thus, linking with public information allows the re-identification of individuals. These attributes are called quasi-identifiers. The identification of which attributes in a given data set are quasi-identifiers depends on the context. Typical examples are date of birth, gender, and address. Less common examples include geographic location and occupation. Besides that, one must also consider the existence of proxy data; i.e. some types of data may be inferred to create a more complete data set, making the re-identification process easier.

Because of previous adversity, identity disclosure is one of the biggest concerns of data privacy in the current information age. An intruder might be internal or external to the organisation with a particular motivation, for instance, a dissatisfied employee, an investigative journalist or even a research team. A motivated intruder can improve their knowledge of private information on observations in available data. Before releasing the data set, a re-identification risk assessment should be carried out to verify the possibility of an intruder linking the information in the data set to an external data set and, consequently, avoiding the exposure of private information of a specific individual.

For this purpose, the uniqueness of the records is a relevant feature. *k*-Anonymity [15] is a commonly used method for enhancing individual privacy which provides the uniqueness of observations. *k*-Anonymity is achieved if, for any individual, there are at least *k*-1 individuals in the data set that share the same properties. The set of these *k* records compose an equivalence class. However, this privacy method has some flaws, such as the intruder can obtain sensitive information about the individual even if the data set satisfies *k*-anonymity. Sensitive information refers to highly critical attributes, usually protected by law and regulations. Examples include religion, sexual orientation, health information and political opinion. The *l*-diversity [16] is a *k*-anonymity extension that measures the diversity of values for each sensitive attribute, allowing to identify possible losses of privacy. A data set satisfies *l*-diversity if for every equivalence class there are at least *l* different values for each of the sensitive attributes.

### Linkability

The effectiveness of a de-identification process can be assessed by the difficulty in record-linkability, within or between databases. The linking process corresponds to the ability to connect or correlate two or more records concerning the same individual or a group of individuals [17]. Before the release of data, it is crucial to verify linkability. An intruder may link the supposedly de-identified data with external information and thus obtain private data about an individual. The intruder can use such private information for malicious purposes, e.g. identity theft [18], frequently used in fraudulent efforts for financial gain, including credit card fraud, tax fraud, utility fraud, lease fraud or even loan fraud.

Record linkage is a technique that attempts to link records on different data sets identifying the presence of the same entity. This process classifies each record pair as a true match based on whether they agree or disagree on the selected attributes. Multiple previous research studies, mainly in the field of health, have demonstrated the effectiveness of this technique [19–21].

## Materials and methods

In this section, we provide an experimental study concerning re-identification risk. To achieve such an objective, we first apply data pre-processing to obtain a richer data set for further analysis. Then, we assess the risk of re-identification for each record. Finally, we link the provided data set and an external data set for demonstration purposes.

### Data preparation

The Direção Geral de Saúde (DGS), the health authority in Portugal, provided a data set of Covid-19 cases from the beginning of January 2020 until mid-April of the same year. This data set has 20.293 records, each corresponding to a single individual. The description of each attribute of such data set follows in Table 1.

**Table 1:** Description of data set with information on Covid-19 cases in Portugal.

Variable	Type	Description
**RecordId**	*String*	Unique case identifier
**Age**	*Numeric*	Age of patient in years as reported in the national system at the time of disease onset
**AgeDay**	*Numeric*	Age of patient in days as reported in the national system for cases < 1 month of age at the time of disease onset
**AgeMonth**	*Numeric*	Age of patient in months as reported in the national system for cases < 2 years of age at the time of disease onset
**DateOfFirstPositiveResultLab**	*Date*	Date when first positive laboratory result became available
**DateOfHospitalisation**	*Date*	Date of Hospitalisation
**DateOfOnset**	*Date*	Date of onset of disease. Not applicable in asymptomatic cases
**Gender**	*String*	Gender of the reported case
**Hospitalisation**	*String*	Admission to hospital
**IntensiveCare**	*String*	Case required care in an intensive care unit
**Outcome**	*String*	Information on the outcome of the case
**PlaceOfInfection**	*String*	The probable place of infection should be provided at the NUTS 3 level
**Precondition**	*String*	Patient’s underlying condition or conditions
**PreconditionOther**	*String*	Details of underlying conditions
**RespSupport**	*String*	Level of respiratory support given to patient

In this period, 2.798 of the cases correspond to individuals at higher risk, i.e. those of advanced age (80+ years). However, the highest number of infected cases corresponds to the age range of 50 to 59 with 3.211 individuals. Of the total number of cases, 216 are in intensive care, and 85 of these need respiratory support. Female individuals are the most affected by the disease corresponding to 11.903 records. The three most prevalent underlying conditions are diabetes, followed by lung problems and cancer. The northern part of the country recorded the most cases. 18.524 individuals remained in treatment for Covid-19, and 1.244 were able to recover, but unfortunately, there are 502 deaths due to this disease.

In order to prepare the data for the application and study of risk measures, it underwent the following transformations:

- The symbol *NA* replaced empty fields;
- The “Precondition” and “PreconditionOther” attributes were disintegrated and transformed into binary attributes to clarify the information on the pathologies of each patient;
- The “Health” and “Pregnant” attributes were created, based on the previously created attributes. They indicate whether a patient is healthy or suffers from some pathology and if the record relates to a pregnancy case, respectively.

### Assessment of re-identification risk

The information provided is at the level of the individual; this type of information is called microdata. Assessment of re-identification risk is crucial in microdata release. Accordingly, we assess individual risk using the *k*-anonymity method.

Given *k*, the number of combinations of a given set of quasi-identifiers in the data set, *fk* is the frequency of the sample records having the same combination *k* of quasi-identifiers. If *fk* = 1, the individual has a unique combination of quasi-identifiers values, so we can *single out* that individual. The rarer a combination of values of the quasi-identifiers, the greater the risk of disclosure of the individual’s identity.

### External data set

We consider personal data de-identified if there is no possibility of re-identification. At this stage, the intention is to reveal with a high level of accuracy the individual’s identity described by a particular record.

For this purpose, we start by acquiring all external information through a single information vector, the media. We carried out such research mostly resorting to two newspapers. It was possible to acquire 68 cases of death by Covid-19 in Portugal on these platforms. Table 2 illustrates the personal information collected from the research.

**Table 2:** Description of external information collected in the media on cases of deaths from Covid-19.

Variable	Type	Description
**Date**	*Date*	Date of death
**Gender**	*String*	Gender of the patient
**Age**	*Numeric*	Age of patient
**Hospital**	*String*	Hospital where the death was declared
**Name**	*String*	Name of the patient
**Address**	*String*	Address of the patient
**DaysHospitalisation**	*String*	Number of days the patient was hospitalised until the day of death
**Diseases**	*String*	Patient’s underlying health conditions
**Profession**	*String*	Profession of the patient
**RetirementHome**	*String*	Retirement home that the patient frequented

Through the address of the individual, we know which NUTS3 region it is likely to belong. Therefore, we created a new attribute with this information to link with the infection site of DGS subset.

Therefore, we use this created data set to obtain more knowledge on the deaths’ records present in the data set released by DGS. The linkage process was performed with *Python Record Linkage Toolkit* [22], which provides robust tools to automate record linkage. This tool uses a distance function to verify the similarity between two strings. The tool was applied using the default parameter values. We compare the external data set with the released data set, and we analyse how many records are coincident.

## Results and discussion

We start by presenting the re-identification risk results throughout the provided data set, considering two attack scenarios. Then we show the re-identification risk results in the subset of deaths considering a realistic scenario and how many cases we can re-identify by linking to the external data set. We also provide an extra analysis of a second data set also released by DGS. Finally, we recommend appropriate transformation techniques for contexts similar to those of the case studied.

### Initial data set

In a first phase, the quasi-identifiers “Age”, “Gender”, “PlaceOfInfection” and “Outcome” were selected. After obtaining the *fk* values for all records, we found that there are 1.413 unique samples for these quasi-identifiers. Thus, by knowing the age, gender, place of infection and also the final result of the case, it can be stated that 6.96% of individuals are identifiable without any doubt.

To ensure that there is no exposure of private information, we need to consider several attack scenarios as we do not know what information the intruder has. For this reason, we carried out a new assessment with the addition of the quasi-identifier “DateOfHospitalisation”. For this scenario, cases with the easiest re-identification correspond to 16.71%. Note that, regarding this quasi-identifier the data set has 17.704 missing data, that lead us to believe that if we had better data quality, the risk would be higher.

Even in cases where the data set satisfies *k*-anonymity, i.e. each combination of quasi-identifiers values appears at least *k* occurrences in the data set, there is a risk that sensitive information can be disclosed. Such risk surges when data contains sensitive information with the same value for all individuals sharing the same quasi-identifiers. *l*-Diversity depends on the number of possible values that the sensitive attribute can take. A unique combination always satisfies *l*-diversity. This measure was applied to the sensitive attributes “Health” and “Pregnant” when *fk* >1. With information about the health status of the patients, 24.34% of them lose privacy. In other words, although it is not known what the “RecordId” of an individual is, it is known with certainty that this individual suffers from some problem. Regarding the pregnancy situation, this attribute presents missing values for male individuals, for children up to 15 years of age and for women over 50 years of age. Nevertheless, women at risk of loss of privacy represent 17.67% of the population.

### Subset of deaths

In this step, we only select observations in which there is a death record, corresponding to 502 cases. The calculation of risk with the *k*-anonymity was performed with the quasi-identifiers “Age”, “Gender” and “PlaceOfInfection”. We conclude that for this subset, 38.24% of individuals are easily identifiable.

In this subset of data, there are no records on the deaths of pregnant women. As such, we only use the sensitive attribute on health status for the calculation of *l*-diversity. We conclude that such information could cause a loss of privacy for 16.14% of individuals.

### Re-identification attack in the subset of deaths

Assuming as probable knowledge data the gender, age and place of infection, and realising the linkage attack, of the 38.24% of easily identifiable individuals, which corresponds to 192 cases in the subset, 6.25% of them are directly identifiable. Table 3 presents the re-identification results for all *fk* in this subset using the record linkage method [23].

**Table 3:** Results of the re-identification attack by linking the subset of deaths with external information of deaths from Covid-19.

**fk**	1	2	3	4	5	6	7
**Certainty in re-identification**	100%	50%	33%	25%	20%	17%	14%
**Frequency**	192	90	93	44	25	30	28
**Identified**	12	26	15	12	3	0	0

Additionally, knowing the sensitive information “Health”, it is confirmed that of the 81 individuals (16.25%) who presented a risk of loss of privacy, 21 of these individuals disclose this information.

We achieved such an outcome by cross-referencing media information. If we would explore other vectors of information, for example, social networks, identification of more individuals and the retrieval of their personal information would be highly likely.

### Aftereffect of adding new data points

To improve progress in finding new responses to the Covid-19 fight, DGS has provided a new data set with records from January to June’s last day (38.546 records). This second version of the data, in addition to new records, contains transformations in some attributes presented in Table 1. Instead of age separated by days, months and years, the age is represented in only one attribute. The attributes concerning the underlying conditions of the patient were disintegrated into new attributes, each one representing a pathology, with the values “Yes”, “No” or “Unk”. The old attribute “Outcome” was replaced by two new date attributes: recovery date and death date. In addition to these transformations, it also contains new attributes with the diagnosis date and discharge date. The remaining attributes of the second data set remained the same as the first version.

Our goal was again to assess the risk of re-identification in this new data set. Suppose the intruder is a laboratory with probable knowledge data on age, gender, place of infection, and diagnosis date. The risk of *single out* taking into account these quasi-identifiers is 53.43%. This high percentage is mainly due to the number of distinct values in the diagnosis date attribute. We point out that this attribute has only four missing values.

As in the first version of the data, we also calculated the risk of re-identification for the deaths subset. Although we do not have the outcome of each case, we have the date of death, and this is relatively easy information for the intruder to acquire. Considering the cases of death, we have a subset with 1.155 records. We have added the attribute about dates of death to the previous set of quasi-identifiers for this subset. Consequently, we have carried out the re-identification risk assessment and found out that 99.65%, corresponding to 1.151 individuals, are directly identifiable. It is highly unlikely that two or more individuals of the same age, gender, place of infection, and diagnosis date died on the same day. Clearly, the combination of this information leads to a high risk of re-identifying individuals in this subset.

Therefore, we must consider some modifications to this second version of the data for the sake of the individual’s privacy. In the following section, we present a short set of recommendations to transform data to protect individuals’ privacy. Such transformations are suitable for both the first and the second version of the data.

### Recommendations

A new level of de-identification may be further considered to protect the privacy of data subjects. The Article 29 Working Party published new guidance on de-identification techniques [17]. Researchers also advise on some measures to preserve patient safety in health data taking into account the risk of re-identification [24, 25].

Regarding our study, so that the individual is not singled out, it can be grouped with other individuals who share the same values. This process is called generalisation, which replaced the specific values with more general values; for example, a patient’s street address could be generalised to the district. Note that generalisation is not the same as aggregation. The information in aggregated data is presented as a summary; for example, if a patient goes to the hospital several times, we can summarise that information with the number of times they go to the hospital.

The generalisation can be applied to the attribute “Age” instead of age in years (31), this can be represented in an age group ([30-35)). In addition to this attribute, the site of infection may also undergo the same process. Reduce the granularity of the sub-region (NUTS3) to the region (NUTS2).

Generalisation is a non-perturbative method. These methods reduce details in the data and also suppress values. However, perturbative methods are also a possibility to ensure the privacy of the data subject. Perturbative methods do not suppress the values in the data set but change the values to limit disclosure risk, creating inaccuracy on the original data. Thus, an intruder is unsure whether or not the correspondence between the disclosed data set and the external is correct. In this sense, noise can be added to the “Age” attribute.

Dates related to an individual may also compromise their privacy. Previously, it was found that adding the attribute “DateOfHospitalisation” (first version of data) to the tuple of quasi-identifiers increased the identifiable cases. For this reason, the date can be replaced by a new date generated using a random shift for each individual, and this shift is applied to all dates. The shift maintains the relative distance between dates. However, attributes about dates, rather than a shift, can also be generalised. For example, instead of presenting the dates in detail, they can be presented in week numbers or the month in which it happened.

In short, we indicate two types of methods to protect the individual’s privacy: perturbative methods and non-perturbative methods. In this study, we only mention generalisation as a non-perturbative method; and noise and the generation of new dates as perturbative methods.

The utility of the data is an essential factor in choosing the type of transformation methods. We must analyse the utility of the data from the perspective of the loss of information caused by the transformation. If necessary, we must consider another type of methods to protect the privacy of individuals.

#### Demonstration of generalisation

Although we suggest different transformation methods to achieve a further protected data set, data protection through generalisation is the most widely referred to in the literature. Therefore, we intend to demonstrate the impact that the generalisation can have in reducing the re-identification risk.

**Fig 1:**
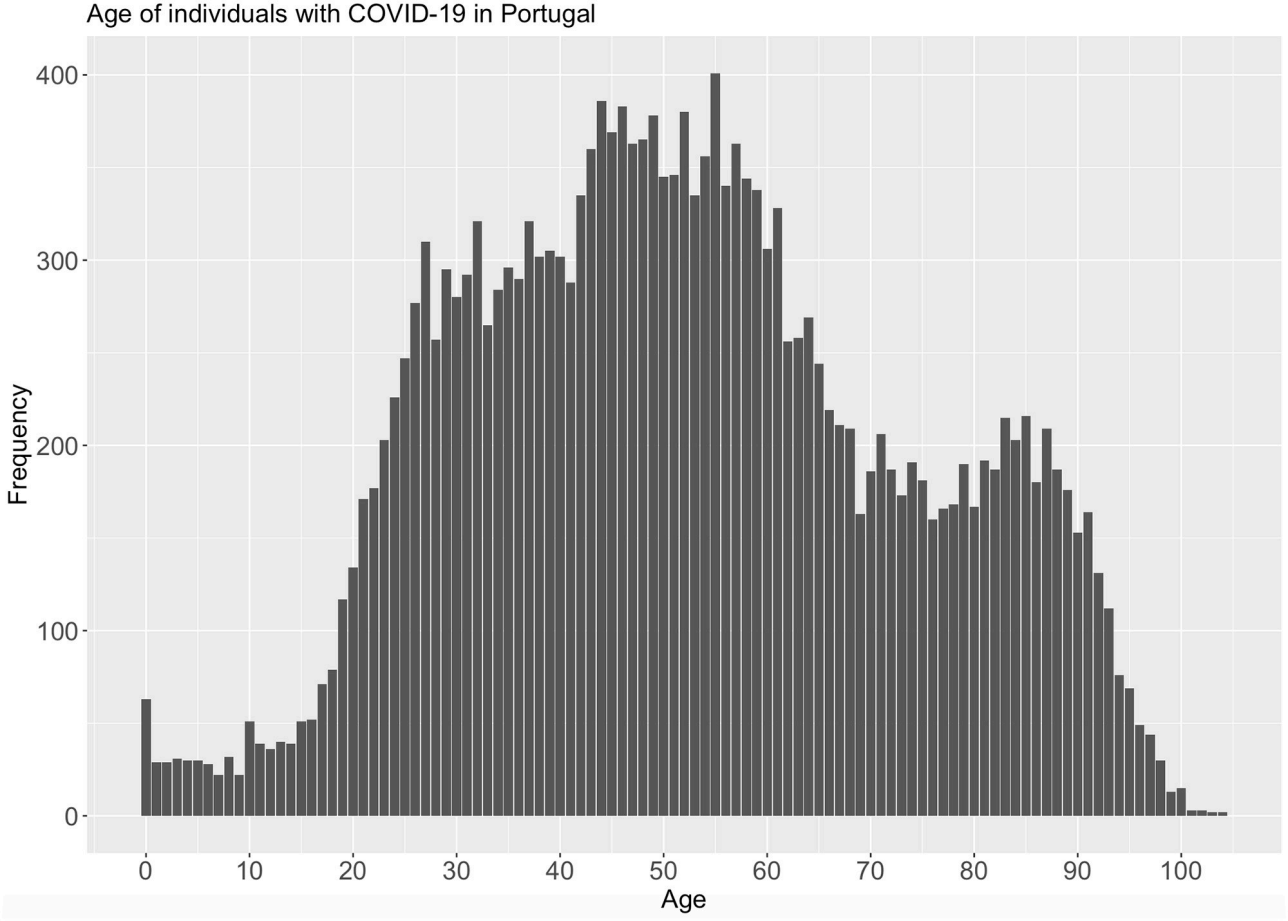
Age attribute distribution in the initial data set.

**Fig 2:**
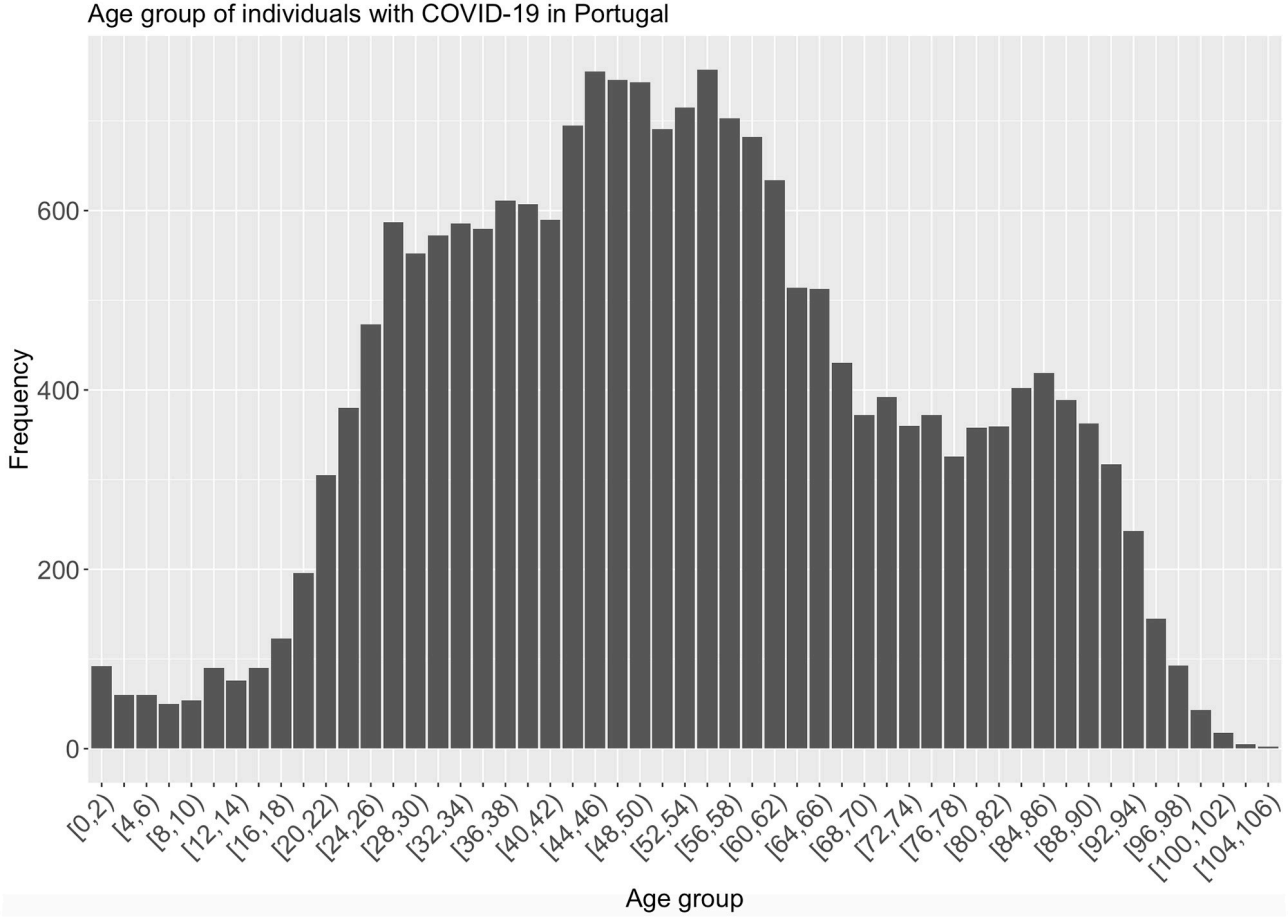
Age attribute distribution after the construction of age intervals.

As mentioned earlier, one of the attributes that can be represented in less specific values is age. In the experience with this attribute, age ranges of size two were created regarding the first version of data. Figs 1 and 2 present the age distribution before and after the application of generalisation technique, respectively.

Given this transformation in the attribute on age, the risk of disclosure was re-analysed. The *fk* was obtained with the same quasi-identifiers (“Age”, “Gender”, “PlaceOfInfection” and “Outcome”). Even considering a short interval of only two years, the number of unique combinations, i.e. the number of easily identified individuals, decreases from 6.96% to 3.97%. Compared to the original data set, this transformation is a considerable improvement concerning data subjects’ privacy.

By analysing the previous figures, we observe that age distribution remains similar after the application of generalisation. Such similarity provides an example of how it is possible to increase individuals’ privacy while maintaining the utility of the data.

## Conclusion

The urgent need to get answers during the Covid-19 pandemic raised several data protection questions. For researchers, data protection has proven to be a challenge at this time [26]. During a public health crisis, it is necessary to assess the balance between personal privacy and public interest without discouraging the need to limit data collection and use. Therefore, it is crucial to design more robust de-identification mechanisms to keep up with the technologies and tools available today.

To judge whether a data set is safe enough for release, it is essential to identify the risks to which the data subject is exposed and project future risks that may occur and identify procedures to minimise and correct these errors. Quantifying the risk of re-identification is not a simple task [27], so it should be a continuous work, with frequent risk assessment.

The guarantee of strong privacy protection requires the transformation of original data, and as a consequence, the utility of data can be reduced. Therefore, data utility and individual privacy are commonly on opposite sides. The more information is removed about an individual, less useful the data may become for statistical analysis or other purposes. Under such circumstances, proposed changes should also be explored from a utility perspective. This process of analysis should be iterative until the best compromise between utility and privacy is reached. However, other privacy-preserving approaches have been proposed in the literature, like homomorphic encryption [28, 29] or differential privacy [30], that may not necessarily hamper the data analysis.
